# The association between resting heart rate and HbA1c-assessed glycemic control in patients with type 2 diabetes in Eastern China

**DOI:** 10.3389/fendo.2025.1478575

**Published:** 2025-06-16

**Authors:** Xiangyu Chen, Feng Lu, Jie Zhang, Xiaofu Du, Chunxiao Xu, Mingbin Liang, Lijin Chen, Jieming Zhong

**Affiliations:** Department of Non-Communicable Disease Control and Prevention, Zhejiang Provincial Center for Disease Control and Prevention, Hangzhou, China

**Keywords:** resting heart rate, glycemic control, cross-sectional study, type 2 diabetes mellitus, autonomic dysfunction

## Abstract

**Objectives:**

This study aimed to explore the association between resting heart rate (RHR) and HbA1c-assessed glycemic control in patients with type 2 diabetes mellitus (T2DM).

**Methods:**

This cross-sectional study was conducted in Zhejiang Province, Eastern China, from March to November 2018. The association between RHR and inadequate glycemic control was analyzed using multivariable logistic regression and restricted cubic spline models. Additionally, a generalized additive model was employed to examine the association between RHR and HbA1c levels.

**Results:**

A total of 1,756 patients with T2DM were included in this study. The prevalence of inadequate glycemic control was 48.92% in this population. After adjusting for age, sex, educational level, body mass index, hypertension, abnormal total cholesterol, abnormal triglyceride, cigarette smoking, and duration of diabetes, when compared to the first quintile of RHR (< 70 beats per minute [bpm]), patients in the second quintile (70–75 bpm), third quintile (76–80 bpm), fourth quintile (81–87 bpm), and fifth quintile (≥88 bpm) had increased risks of inadequate glycemic control, the adjusted odds ratios (ORs) with 95% confidence intervals (CIs) were: 1.11 (0.82-1.49), 1.50 (1.10-2.06), 1.70 (1.25-2.31), and 2.14 (1.56-2.94), respectively. When RHR was treated as a continuous variable, each 10 bpm increase was associated with a 27% higher risk of inadequate glycemic control (OR: 1.27; 95% CI: 1.16-1.39). Moreover, HbA1c levels were positively correlated with increasing RHR in this population (Spearman correlation coefficient=0.15, P<0.001). Subgroup analyses confirmed that the association between elevated RHR and inadequate glycemic control persisted across all key demographic strata (all p < 0.05). Notably, BMI significantly modified this relationship (p for interaction < 0.05), with a more pronounced effect observed in individuals with higher BMI.

**Conclusions:**

Elevated RHR is associated with inadequate glycemic control and higher HbA1c levels. Our findings suggest a potential bidirectional relationship between RHR and glycemic control in T2DM patients. These results may contribute to individualized clinical management and inform targeted public health strategies aimed at early identification and intervention in high-risk T2DM populations.

## Introduction

Diabetes mellitus, characterized by chronically elevated blood glucose levels, has reached alarming prevalence levels, emerging as a significant public health challenge in China (1). This poses substantial threats to societal well-being and economic stability. China has witnessed one of the most significant increases in diabetes prevalence worldwide, primarily driven by type 2 diabetes (T2DM) (2). T2DM can lead to various complications, such as retinopathy, kidney disease, and atherosclerosis (3). Achieving optimal glycemic control not only mitigates the risk of acute and chronic complications but also enhances the overall quality of life for individuals living with diabetes. Several major studies, including the Veterans Affairs Diabetes Trial and the United Kingdom Prospective Diabetes Study, have demonstrated the critical role of maintaining appropriate glycemic levels in individuals with T2DM (4, 5).

Resting heart rate (RHR) serves as a sensitive indicator of autonomic nervous system activity, reflecting the balance between sympathetic and parasympathetic functions (6). Research has shown that heightened sympathetic activity not only raises RHR but also exacerbates insulin resistance (IR) (7, 8). Numerous epidemiological studies have consistently linked elevated RHR with an increased risk of cardiovascular disease and all-cause mortality across both sexes (9–11). Additionally, RHR has been identified as a significant factor in the context of T2DM. High RHR is associated with an increased risk of developing T2DM, as evidenced by various epidemiological studies. For instance, the Australian Diabetes Obesity and Lifestyle Study found that elevated RHR correlates with an increased risk of diabetes over a 5-year span, particularly among non-obese males (12). Similarly, the results from the Chicago Heart Association Detection Project in Industry Study revealed a positive correlation between middle-age RHR and subsequent diagnoses of diabetes and diabetes-related mortality in later years (13). Moreover, RHR has been significantly associated with incident prediabetes (14). However, comprehensive studies exploring the association between RHR and glycemic control in T2DM patients remain limited.

Investigating the potential correlation between RHR and glycemic control is clinically significant, offering opportunities for innovative therapeutic strategies in diabetes management. This study examined the correlation between RHR and glycemic control among individuals with T2DM, utilizing data from the Zhejiang Provincial Diabetic Complications Study. The results are expected to provide valuable insights for developing strategies to optimize blood glucose levels, thereby reducing the risk of complications in the T2DM population.

## Methods

### Study design and population

This study, conducted from March to November 2018, was part of the China National Diabetic Complications Study. It sought to identify the prevalence and risk factors associated with diabetic complications among T2DM patients in Zhejiang Province, Eastern China (15). Detailed information about the study’s design, methodologies, and participant criteria is available in the published data resource profile (16). The study targeted eligible individuals aged 18 years or older with T2DM who had resided in the survey areas for at least six months within the past year.

A multi-stage random sampling technique was utilized for the study. Initially, 2 districts and 2 counties within Zhejiang Province were randomly selected. Subsequently, 4 streets or towns from each of these districts and counties were chosen at random. The final step involved randomly selecting 120 T2DM patients from each street or town, stratified by sex and age, resulting in a total of 1,920 participants. Each participant underwent a comprehensive physical examination, fasting blood tests, and a detailed face-to-face questionnaire survey (15).

The research was approved by the ethics committee (Approval No: 2018-010) and registered with the Chinese Clinical Trial Registry (ChiCTR1800014432). All participants provided written informed consent (15), and the study adhered strictly to the Declaration of Helsinki.

### Data collection and measurements

The questionnaire survey was carried out by personnel from the local centers for disease control and prevention and local primary healthcare facilities. These trained individuals gathered participant information regarding demographics and health behaviors through direct oral questionnaires. Physical examinations were performed at primary healthcare centers by experienced healthcare providers and included measurements of height, weight, waist circumference, and blood pressure (BP). Height was measured to the nearest 0.1 cm using a TZG stadiometer, and weight was recorded to the nearest 0.1 kg using an HD-390 scale (TANITA, Japan). Blood pressure and resting heart rate (RHR) were measured three times at one-minute intervals using an HBP-1300 electronic monitor (OMRON, Japan), with the average of the three readings used in analysis. Fasting venous blood samples were collected to assess multiple biochemical indicators, including fasting plasma glucose (FPG), hemoglobin A1c (HbA1c), total cholesterol (TC), triglycerides (TG), high-density lipoprotein cholesterol (HDL-C), and low-density lipoprotein cholesterol (LDL-C). Lipid parameters (TC, TG, HDL-C, LDL-C) were measured using enzymatic methods on a Roche cobas c701 automated analyzer (Roche, Switzerland). FPG was assessed using the hexokinase method, and HbA1c was measured via high-performance liquid chromatography using a Hemoglobin Analyzer (Bio-Rad, USA).

### Definition of the variables

The primary outcome variable in this study was inadequate glycemic control, characterized by an HbA1c level of 7.0% or higher. Hypertension was defined as having a systolic blood pressure (SBP) of 140 mmHg or more and/or a diastolic blood pressure (DBP) of 90 mmHg or more, in addition to a self-reported diagnosis of hypertension. Age was categorized into three groups: young adults (18 to 44 years), middle-aged adults (45 to 59 years), and older adults (60 years and older). BMI was categorized into two groups: <24 kg/m^2^ and ≥24 kg/m^2^. Educational attainment was divided into three levels: secondary school or less, senior high school, and college or higher. Participants were classified based on their residence as either urban or rural. Smoking status was determined by whether participants smoked cigarettes daily or occasionally, while alcohol consumption was identified by any alcohol intake within the past thirty days. The duration of diabetes was categorized into four groups: 5 years or less, 6–10 years, 11–15 years, and more than 15 years. Abnormal lipid levels were defined as follows: TC ≥ 6.22 mmol/L, TG ≥ 2.26 mmol/L, LDL-C ≥ 4.14 mmol/L, and HDL-C < 1.04 mmol/L.

### Statistical analysis

Continuous variables were expressed as mean ± standard deviation (for normally distributed data) or median (interquartile range [IQR]) (for non-normally distributed data). Between-group comparisons were performed using independent t-tests or ANOVA for normally distributed data, and Wilcoxon rank-sum or Kruskal-Wallis tests for non-normally distributed data. Categorical variables were presented as frequencies (percentages) and compared using χ^2^ tests. The RHR-HbA1c relationship was assessed through generalized additive models (GAMs) and Spearman correlation (ρ). Unconditional multivariable logistic regression models were applied to pinpoint factors associated with inadequate glycemic control, with covariates selected through backward elimination. Three models were constructed: Model 1 (unadjusted), Model 2 (age and sex adjusted), and Model 3 (fully adjusted for age, sex, education, BMI, hypertension, unfavorable lipid profile [abnormal TC/TG], smoking, and diabetes duration). Restricted cubic splines (RCS) characterized the dose-response relationship between RHR and inadequate glycemic control. Furthermore, subgroup analyses were performed based on key demographic variables to assess potential effect modification by age, sex, BMI, smoking status, and alcohol consumption. All analyses were performed using SAS 9.4 (SAS Institute), with two-tailed p<0.05 considered statistically significant.

## Results

### Basic characteristics of the participants

The present study analyzed data from 1,756 participants who provided complete research information. **Table 1** details the general characteristics of participants categorized by glycemic control status. Among the total, 876 (49.89%) were male, with an average age of 57.23 ± 10.15 years and mean BMI of 24.76 ± 3.43 kg/m². The median RHR was 77 bpm (IQR: 71-85), and diabetes duration varied: 836 (47.61%) had a duration of ≤5 years, 491 (27.96%) had 6–10 years, 236 (13.44%) had 11–15 years, and 193 (10.99%) had >15 years. Inadequate glycemic control affected 859 (48.92%) participants. Additionally, 1,099 (62.59%) had hypertension, while 436 (24.83%) and 646 (36.79%) reported cigarette smoking and alcohol consumption, respectively. **Table 1** also highlights that participants with inadequate glycemic control had higher BMI, RHR, DBP, TG, TC, LDL-C, and FPG levels, along with a longer duration of diabetes compared to those with adequate glycemic control (all P < 0.05). **Table 2** stratifies baseline characteristics by RHR quintiles, revealing significant differences in age, sex distribution, DBP, TG, TC, FPG, HbA1c, prevalence of inadequate glycemic control, and cigarette smoking across RHR categories (all P < 0.05).

**Table 1:** Basic characteristics of the participants according to different glycemic control status (n=1,756).

Characteristics	Overall (n=1,756)	Group without inadequate glycemic control (n=897)	Group with inadequate glycemic control (n=859)	t/χ^2^/z	p
Age (years) [means ± SD]	57.23 ± 10.15	57.74 ± 10.11	56.70 ± 10.18	2.16 ^a^	0.031
Sex,n (%)				0.38^b^	0.536
Male	876 (49.89)	441 (49.16)	435 (50.64)		
Female	880 (50.11)	456 (50.84)	424 (49.36)		
Educational level,n (%)				4.01 ^b^	0.134
Secondary school and lower	1,541 (87.76)	780 (86.96)	761 (88.59)		
Senior high school	171 (9.74)	88 (9.81)	83 (9.66)		
College or above	44 (2.50)	29 (3.23)	15 (1.75)		
Residence,n (%)				0.04 ^b^	0.851
Rural	875 (49.83)	445 (49.61)	430 (50.06)		
Urban	881 (50.17)	452 (50.39)	429 (49.94)		
BMI (kg/m^2^) [means ± SD]	24.76 ± 3.43	24.50 ± 3.38	25.03 ± 3.47	-3.23 ^a^	0.001
RHR (bpm) [median (IQR)]	77 (71–85)	76 (70–83)	79 (72–87)	32.61^c^	<0.001
SBP (mmHg) [means ± SD]	136.45 ± 18.71	133.06 ± 16.90	135.32 ± 18.11	-2.70 ^a^	0.007
DBP (mmHg) [means ± SD]	78.39 ± 10.68	76.53 ± 9.83	78.38 ± 10.25	-3.84 ^a^	<0.001
Hypertension,n (%)	1,099 (62.59)	569 (63.43)	530 (61.70)	0.56^b^	0.453
TG (mmol/L) [median (IQR)]	1.60 (1.12-2.42)	1.49 (1.08-2.22)	1.74 (1.18-2.65)	26.37^c^	<0.001
TC (mmol/L) [means ± SD]	4.65 ± 1.07	4.50 ± 0.98	4.83 ± 1.14	-6.37 ^a^	<0.001
HDL-C (mmol/L) [means ± SD]	1.25 ± 0.36	1.27 ± 0.36	1.23 ± 0.35	2.52 ^a^	0.012
LDL-C (mmol/L) [means ± SD]	2.73 ± 0.90	2.62 ± 0.83	2.85 ± 0.96	-5.28^a^	<0.001
FPG (mmol/L) [means ± SD]	7.94 ± 2.58	6.65 ± 1.31	9.29 ± 2.87	-24.56 ^a^	<0.001
HbA1c (%) [means ± SD]	7.27 ± 1.49	6.20 ± 0.49	8.39 ± 1.35	-45.12 ^a^	<0.001
Smoking,n (%)	436 (24.83)	205 (22.85)	231 (26.89)	3.83^b^	0.050
Drinking,n (%)	646 (36.79)	334 (37.24)	312 (36.32)	0.16^b^	0.691
Duration of diabetes (years),n (%)				35.44^b^	<0.001
<=5	836 (47.61)	478 (53.29)	358 (41.68)		
6-10	491 (27.96)	249 (27.76)	242 (28.17)		
11-15	236 (13.44)	97 (10.81)	139 (16.18)		
>15	193 (10.99)	73 (8.14)	120 (13.97)		

^a^Student’s t-test; ^b^Chi-square test; ^c^Wilcoxon rank-sum test.

BMI, body mass index; RHR, resting heart rate; bpm, beats per minute; SBP, systolic blood pressure; DBP, diastolic blood pressure; TG, triglycerides; TC, total cholesterol; HDL-C, high density lipoprotein-cholesterol; LDL-C, low density lipoprotein-cholesterol; FPG, fasting plasma glucose.

**Table 2:** Baseline characteristics according to the resting heart rate quintiles (n=1,756).

Characteristics	Q1 (n=365)	Q2 (n=384)	Q3 (n=312)	Q4 (n=351)	Q5 (n=344)	p
RHR (bpm) Median (range)	65 (49–69)	73 (70–75)	78 (76–80)	84 (81–87)	94 (88–144)	
Age (years) [means ± SD]	59.57 ± 8.49	58.09 ± 9.66	57.55 ± 9.58	55.99 ± 10.84	54.75 ± 11.36	<0.001^a^
Sex, n (%)						<0.001^b^
Female	147 (40.27)	191 (49.74)	158 (50.64)	188 (53.56)	196 (56.98)	
Male	218 (59.73)	193 (50.26)	154 (49.36)	163 (46.44)	148 (43.02)	
BMI (kg/m^2^) [means ± SD]	24.68 ± 3.06	24.86 ± 2.97	24.86 ± 3.16	24.82 ± 3.86	24.59 ± 4.03	0.570^a^
SBP (mmHg) [means ± SD]	133.73 ± 17.9	133.24 ± 16.13	134.52 ± 17.47	133.53 ± 18.07	136.01 ± 18.09	0.295^a^
DBP (mmHg) [means ± SD]	74.02 ± 9.49	75.75 ± 9.01	78.62 ± 10.2	77.95 ± 9.93	81.35 ± 10.32	<0.001^a^
Inadequate glycemic control,n(%)	148 (40.55)	163 (42.45)	157 (50.32)	190 (54.13)	201 (58.43)	<0.001^b^
Hypertension,n(%)	223 (61.10)	234 (60.94)	195 (62.50)	218 (62.11)	229 (66.57)	0.532^b^
TG (mmol/L) [median(IQR)]	1.51 (1.07-2.24)	1.52 (1.07-2.26)	1.69 (1.15-2.63)	1.65 (1.16-2.50)	1.66 (1.15-2.52)	0.017^c^
TC (mmol/L) [means ± SD]	4.52 ± 1.02	4.66 ± 0.96	4.64 ± 0.86	4.71 ± 1.27	4.78 ± 1.18	0.046^a^
HDL-C (mmol/L) [means ± SD]	1.21 ± 0.34	1.27 ± 0.33	1.24 ± 0.34	1.24 ± 0.38	1.28 ± 0.4	0.075^a^
LDL-C (mmol/L) [means ± SD]	2.68 ± 0.89	2.79 ± 0.88	2.72 ± 0.78	2.72 ± 0.98	2.76 ± 0.94	0.498^a^
FPG (mmol/L) [means ± SD]	7.33 ± 2.18	7.57 ± 2.12	7.81 ± 2.14	8.21 ± 2.76	8.86 ± 3.25	<0.001^a^
HbA1c(%) [means ± SD]	7.00 ± 1.26	7.02 ± 1.2	7.22 ± 1.28	7.44 ± 1.61	7.71 ± 1.88	<0.001^a^
Smoking, n (%)	113 (30.96)	100 (26.04)	71 (22.76)	89 (25.36)	63 (18.31)	0.003^b^
Drinking, n (%)	143 (39.18)	152 (39.58)	120 (38.46)	123 (35.04)	108 (31.40)	0.120^b^
Duration of diabetes (years), n (%)						0.725^b^
<=5	171 (46.85)	194 (50.52)	153 (49.04)	160 (45.58)	158 (45.93)	
6-10	113 (30.96)	103 (26.82)	77 (24.68)	99 (28.21)	99 (28.78)	
11-15	41 (11.23)	44 (11.46)	48 (15.38)	51 (14.53)	52 (15.12)	
>15	40 (10.96)	43 (11.20)	34 (10.90)	41 (11.68)	35 (10.17)	

^a^ANOVA; ^b^Chi-square test; ^c^Kruskal–Waills test.

RHR, resting heart rate; bpm, beats per minute; BMI, body mass index; SBP, systolic blood pressure; DBP, diastolic blood pressure; TG, triglycerides; TC, total cholesterol; HDL-C, high density lipoprotein-cholesterol; LDL-C, low density lipoprotein-cholesterol; FPG, fasting plasma glucose.

### Multivariable regression analysis of RHR quintiles in relation to inadequate glycemic control

A multivariable logistic regression model (**Table 3**) assessed the odds ratios (ORs) and 95% confidence intervals (CIs) for the association of RHR with inadequate glycemic control across RHR quintiles, using the first quintile (<70 bpm) as the reference. In the crude model, compared to the first quintile, the ORs for the second (70–75 bpm), third (76–80 bpm), fourth (81–87 bpm), and fifth (≥88 bpm) quintiles were 1.08 (95% CI: 0.81-1.45), 1.49 (95% CI: 1.10-2.01), 1.73 (95% CI: 1.29-2.33), and 2.06 (95% CI: 1.53-2.78), respectively. Adjusting for age and sex yielded similar ORs. Further adjustments for age, sex, educational level, BMI, hypertension, abnormal TC, abnormal TG, cigarette smoking, and duration of diabetes resulted in ORs of 1.11 (95% CI: 0.82-1.49), 1.50 (95% CI: 1.10-2.06), 1.70 (95% CI: 1.25-2.31), and 2.14 (95% CI: 1.56-2.94). Trends across quintiles were significant in all models (all P < 0.001). Additionally, treating RHR as a continuous variable, each 10 bpm increase was associated with a 27% higher risk of inadequate glycemic control in the fully adjusted model [OR: 1.27 (95% CI: 1.16-1.39)].

**Table 3:** Multivariable regression analysis of RHR quintiles in relation to inadequate glycemic control (n=1,756).

Characteristics	Model 1		Model 2		Model 3	
	OR (95%CI)	p	OR (95%CI)	p	OR (95%CI)	p
RHR quintile						
Q1	1.00 (ref)		1.00 (ref)		1.00 (ref)	
Q2	1.08 (0.81-1.45)	0.598	1.08 (0.81-1.45)	0.601	1.11 (0.82-1.49)	0.510
Q3	1.49 (1.10-2.01)	0.011	1.49 (1.10-2.02)	0.011	1.50 (1.10-2.06)	0.012
Q4	1.73 (1.29-2.33)	<0.001	1.72 (1.28-2.33)	<0.001	1.70 (1.25-2.31)	<0.001
Q5	2.06 (1.53-2.78)	<0.001	2.06 (1.52-2.80)	<0.001	2.14 (1.56-2.94)	<0.001
P for trend		<0.001		<0.001		<0.001
Increase of 10 bpm	1.26 (1.16-1.38)	<0.001	1.26 (1.16-1.37)	<0.001	1.27 (1.16-1.39)	<0.001

RHR, resting heart rate; OR, odds ratio; CI, confidence interval, bpm, beats per minute.

Model 1: unadjusted any covariate; Model 2: adjusted for age, sex; Model 3: adjusted for age, sex, educational level, body mass index, hypertension, total cholesterol abnormal, triglyceride abnormal, cigarette smoking, and diabetes duration.

### GAM analysis of RHR with HbA1c

**Figure 1:**
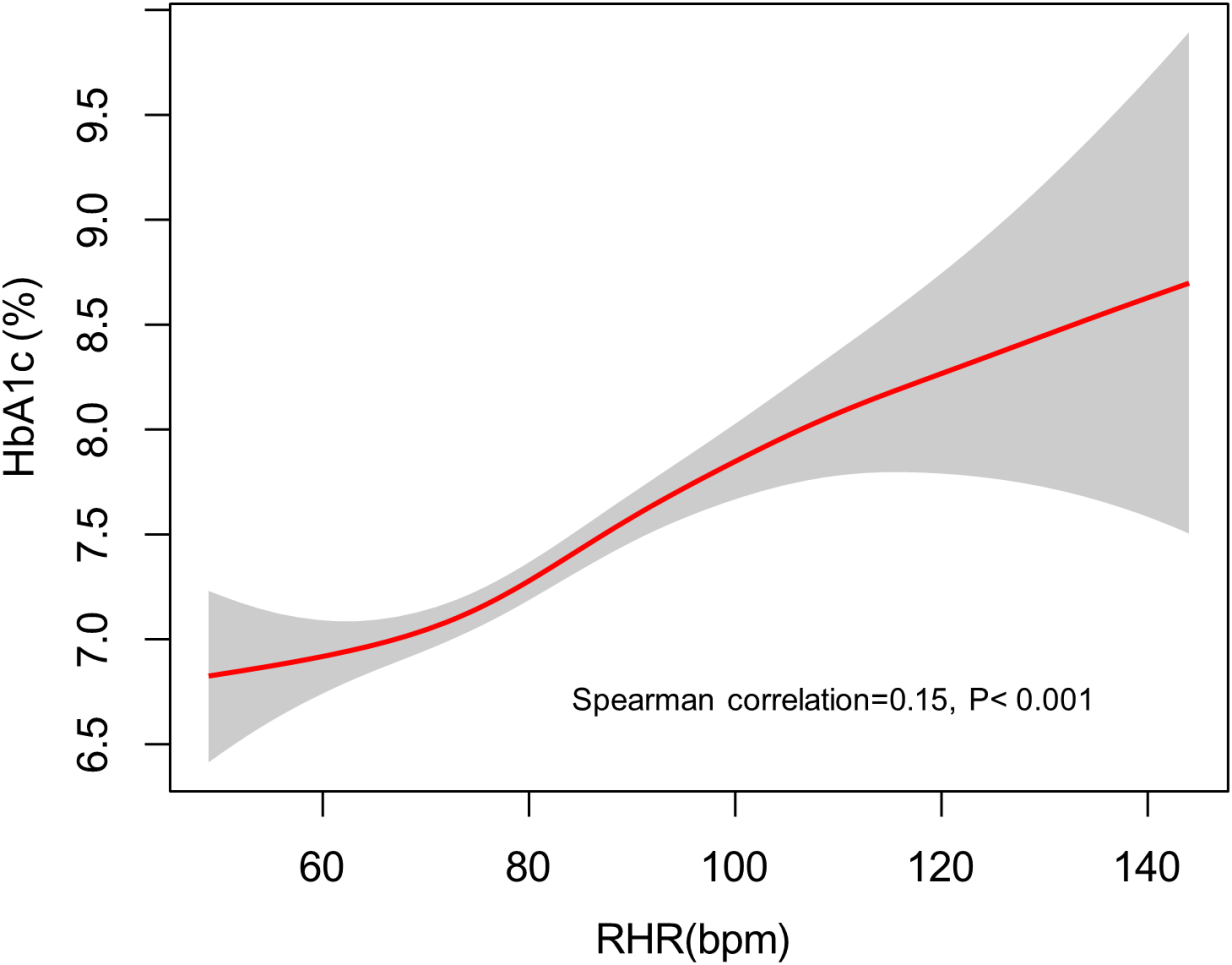
The fitted curve illustrating the association between RHR and elevated HbA1c levels, generated using a generalized additive model. RHR, resting heart rate; HbA1c, hemoglobin A1c.

As depicted in **Figure 1**, a statistically significant positive association was observed between RHR and HbA1c levels (Spearman’s ρ = 0.15, p < 0.001). The nonparametric smoothing curve revealed a consistent monotonic increase, indicating that elevated RHR values correlate with progressively higher HbA1c levels in this population.

### RCS analysis of the association between RHR and inadequate glycemic control

**Figure 2:**
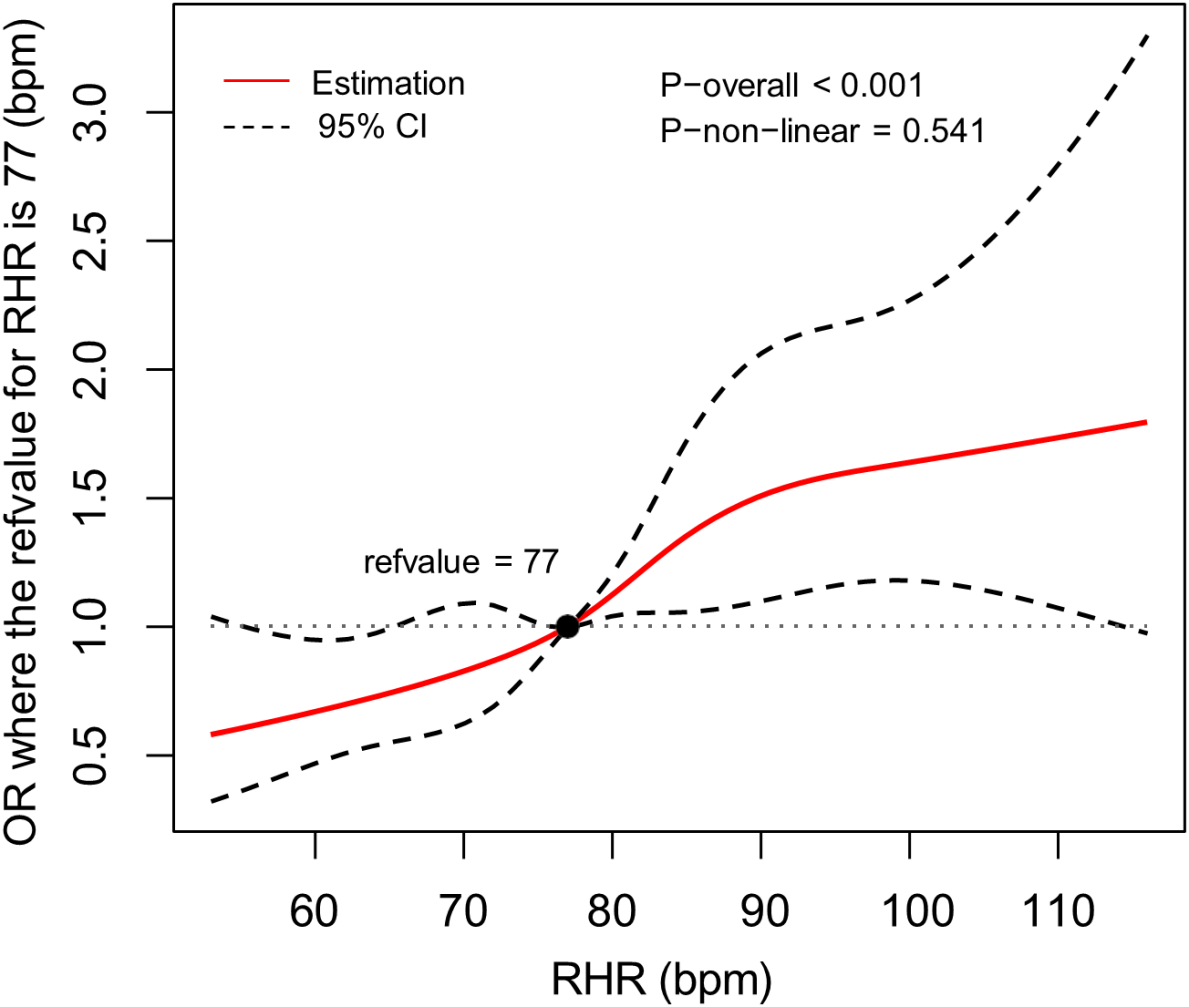
The association between RHR and the risk of inadequate glycemic control, allowing for nonlinear effects, with 95%CI. The model displays ORs relative to the median RHR, set at 77 bpm, adjusting for age, sex, educational level, body mass index, hypertension, total cholesterol abnormal, triglyceride abnormal, cigarette smoking and diabetes duration. RHR, resting heart rate; CI, confidence interval; OR, odds ratio; bpm, beats per minute.

We further employed RCS regression models to examine the dose-response relationship between RHR and inadequate glycemic control risk in patients with T2DM, with comprehensive adjustment for potential confounders including age, sex, education level, BMI, hypertension status, unfavorable lipid profile (abnormal TC and TG levels), smoking status, and diabetes duration. The RCS analysis revealed a significant linear association (P for nonlinearity = 0.541), demonstrating a progressive increase in inadequate glycemic control risk with elevating RHR levels (**Figure 2**).

### Subgroup analyses

**Figure 3:**
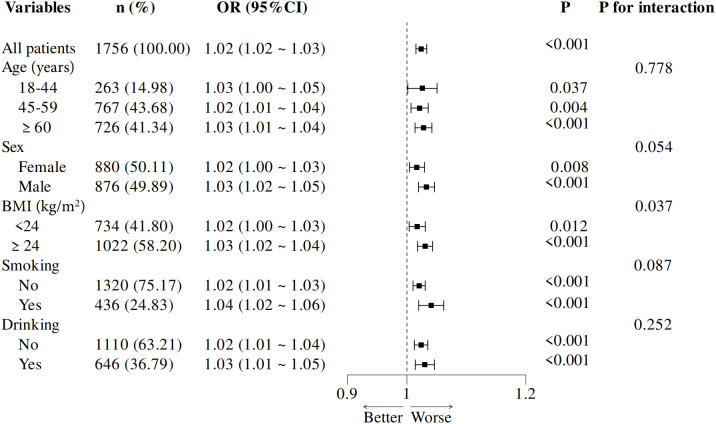
Subgroup analysis of adjusted odds ratios for RHR and inadequate glycemic control. Adjustments were made for age, sex, educational level, body mass index, hypertension, total cholesterol abnormal, triglyceride abnormal, cigarette smoking and diabetes duration. Each subgroup analysis excluded adjustments for the variable defining the subgroup. OR, odds ratio; BMI, body mass index, RHR, resting heart rate.

Subgroup analyses were performed based on key demographic variables, including age (18–44, 45–59, and ≥60 years), sex (male, female), BMI (≥24 kg/m^2^ and <24 kg/m^2^), smoking and drinking status (yes, no) (**Figure 3**). Statistically significant associations were observed across all subgroups (p < 0.05 for each). Notably, participants with a BMI ≥24 kg/m^2^ exhibited a higher OR than those with a BMI <24 kg/m^2^, suggesting a stronger link between RHR and inadequate glycemic control in individuals with higher BMI. These findings imply that BMI may act as an effect modifier in this association, with a more pronounced effect in individuals with higher BMI.

## Discussion

T2DM is a prevalent chronic disease with complex pathophysiology, and its prevention and management, particularly the achievement of adequate glycemic control, remain a significant clinical challenge (17). In the present study, the prevalence of adequate glycemic control among T2DM patients in Zhejiang Province in 2018 was 51.08%, which is notably higher than the national average of 39.70% in China (18), and the rate in Anhui Province (22.97%) (19), and is comparable to those reported in Brazil (51%) (20) and Denmark (49%) (21). Despite this, the large absolute number of individuals with poor glycemic control in Zhejiang reflects a substantial public health burden.

We observed a positive association between elevated RHR and the risk of inadequate glycemic control. After adjustment for major covariates, individuals with an RHR ≥88 bpm had a 2.14-fold increased risk of poor glycemic control compared to those with an RHR <77 bpm. Each 10 bpm increment in RHR was associated with a 27% higher risk. Subgroup analyses further revealed that this association was more pronounced among participants with a BMI ≥24 kg/m^2^, suggesting that BMI modifies the effect of RHR on glycemic control. These findings support RHR as a potential marker for identifying patients at higher risk of metabolic dysregulation, particularly in those with excess body weight.

Although several studies have examined the relationship between RHR and T2DM, their results are not directly comparable to ours due to variations in study design, settings, outcome measures, adjustment variables, and other methodological factors. However, the results still hold some reference value. Echoing our findings, a prospective cohort study revealed that individuals in the highest RHR categories faced a roughly 70% higher risk of developing T2DM compared to those in the lowest categories. Furthermore, each 10 bpm increase in RHR was associated with a 19% elevated risk (22). In a meta-analysis incorporating the aforementioned study (22) and 13 other prospective cohort studies, a positive correlation between RHR and T2DM risk was identified. The summary relative risk per 10 bpm increment was 1.17 (95% CI, 1.09–1.26), while the summary RR for the highest versus lowest RHR categories was 1.44 (1.20–1.74) (22). Similarly, several recent cohort studies conducted in Asian countries also reported findings consistent with these results (6, 23, 24).

Furthermore, our study evaluated the association of HbA1c levels with RHR. Few studies have explored the relationship between glycemic levels and RHR in individuals with diabetes. The most extensive data come from the study by Paterson et al. (25), particularly among participants with type 1 diabetes. In this cohort, intensive treatment targeting HbA1c levels <6.0% resulted in a notably lower RHR over a decade following random assignment in the study (25). This reduction persisted as HbA1c levels converged across initial treatment groups. However, our findings raise the possibility that the relationship may be bidirectional, specifically, higher RHR was associated with elevated HbA1c levels, even after adjustment for key confounders. This suggests that elevated RHR may not only be a consequence of poor glycemic control but may also contribute to it through physiological mechanisms such as increased sympathetic tone and autonomic dysfunction (AD).

The biological basis for the observed correlation between RHR and HbA1c remains largely elusive; nevertheless, findings from prior research indicate that AD may provide an explanation (26, 27). The autonomic nervous system, which encompasses both sympathetic and parasympathetic components, regulates RHR. Thus RHR is an indicator of autonomic activity, elevated RHR often indicates reduced parasympathetic tone or heightened sympathetic activity (27–29). AD is a well-established complication of diabetes, and heart rate variability (HRV) is a relatively simple and non-invasive marker for detecting its early manifestations in patients with T2DM. Alterations in HRV, particularly reductions in time domain and frequency domain indices, have been consistently observed in individuals with type 1 diabetes or T2DM (30, 31). For example, Hajdu et al. demonstrated that HbA1c levels were independent predictors of multiple HRV parameters in individuals with type 1 diabetes, underscoring the importance of glycemic control in maintaining autonomic balance (30). Although their study focused on type 1 diabetes, the implications are relevant to T2DM, where subclinical AD is also highly prevalent. AD may influence glucose regulation through several mechanisms: (1) diminished insulin production, (2) decreased glucose uptake by skeletal muscles due to vasoconstriction, and (3) increased insulin resistance in skeletal muscle cells, stimulated by the renin-angiotensin-aldosterone system(RAAS) (22, 32). Notably, persistent sympathetic overactivity has been associated with obesity, hypertension, and metabolic syndrome, all of which contribute to the development of T2DM due to elevated inflammatory states (33). Conversely, IR and hyperinsulinemia can provoke sympathetic overactivity, contributing to cardiac autonomic dysfunction (34). Additionally, a lower RHR is suggested as a potential indicator of superior cardiorespiratory fitness, which may offer protection against T2DM (35). Moreover, genetic studies have revealed causal links between RHR and T2DM (36). However, further research is necessary to fully elucidate this intricate relationship.

The stronger association between RHR and poor glycemic control observed in individuals with BMI ≥24 kg/m^2^ may reflect the compounding effects of obesity-related metabolic disturbances and autonomic dysregulation. Visceral adiposity is associated with increased secretion of adipokines and pro-inflammatory cytokines (37), which enhance sympathetic activation and impair insulin signaling (38). Individuals with higher BMI may also experience more severe AD, further exacerbating insulin resistance and β-cell dysfunction (39). Thus, BMI may act as an effect modifier by amplifying the metabolic consequences of elevated RHR, resulting in poorer glycemic outcomes.

Taken together, these findings highlight the potential utility of RHR as a simple clinical indicator of suboptimal glycemic control in T2DM, particularly among overweight or obese individuals. Clinically, this underscores the need for integrated management strategies targeting both metabolic and autonomic dysfunction. Interventions such as physical activity, which improve both HRV and insulin sensitivity, may be particularly beneficial. Additionally, incorporating HRV monitoring into routine clinical practice could enhance risk stratification and support individualized treatment approaches.

## Limitation

Several important limitations should be considered when interpreting our findings. First, the cross-sectional nature of our study design prevents us from establishing causal relationships between RHR and glycemic control. Second, while HbA1c is a clinically useful measure of recent glycemic control, it does not reflect long-term glycemic variability or the cumulative burden of hyperglycemia that may contribute to microvascular complications. Third, our single-center study population from Eastern China may limit generalizability to other populations. Finally, we were unable to account for potential confounding by antihypertensive medications (particularly β-blockers) due to limited medication data. Future studies would benefit from longitudinal designs to assess temporal relationships and incorporation of additional glycemic measures (e.g., continuous glucose monitoring) and complication staging.

## Conclusion

In conclusion, our study indicated that elevated RHR is associated with inadequate glycemic control, as well as higher HbA1c levels among patients with T2DM. The potential bidirectional relationship between RHR and HbA1c, along with the effect-modifying role of BMI, highlights the complex interaction between autonomic function and metabolic regulation. These findings have important implications not only for individualized clinical management but also for the development of targeted public health strategies aimed at early identification and intervention in high-risk T2DM populations.

## Data Availability

The raw data supporting the conclusions of this article will be made available by the authors, without undue reservation.
